# Long non-coding RNAs in colorectal cancer

**DOI:** 10.18632/oncotarget.6446

**Published:** 2015-12-02

**Authors:** Xia Xie, Bo Tang, Yu-Feng Xiao, Rui Xie, Bo-Sheng Li, Hui Dong, Jian-Yun Zhou, Shi-Ming Yang

**Affiliations:** ^1^ Department of Gastroenterology, Xinqiao Hospital, Third Military Medical University, Chongqing, P.R. China; ^2^ Division of Gastroenterology, Department of Medicine, School of Medicine, University of California, San Diego, La Jolla, California, USA

**Keywords:** colorectal cancer, long non-coding RNAs, epigenetic modifications, pseudogenes, X-inactive-specific transcript

## Abstract

Colorectal cancer (CRC) is one of the leading causes of cancer-related death worldwide. Despite substantial progress in understanding the molecular mechanisms and treatment of CRC in recent years, the overall survival rate of CRC patients has not improved dramatically. The development of CRC is multifactor and multistep processes, in which abnormal gene expression may play an important role. With the advance of human tumor molecular biology, a series of studies have highlighted the role of long non-coding RNAs (lncRNAs) in the development of CRC. CRC-related lncRNAs have been demonstrated to regulate the genes by various mechanisms, including epigenetic modifications, lncRNA-miRNA and lncRNA-protein interactions, and by their actions as miRNA precursors or pseudogenes. Since some lncRNAs can be detected in human body fluid and have good specificity and accessibility, they have been suggested to be used as novel potential biomarkers for CRC diagnosis and prognosis as well as in the prediction of the response to therapy. Therefore, in this review, we will focus on lncRNAs in CRC development, the mechanisms and biomarkers of lncRNAs in CRC.

## INTRODUCTION

In developed countries, nearly 100 million people suffer from CRC each year, and the mortality rate approaches 33% [1]. Almost 50% of patients exhibit recurrence and die within 5 years, even when there is a curative intent for diagnosis or treatment [2]. Therefore, there is an urgent need to improve the early diagnosis and treatment of CRC. The genesis of CRC involves multi-factorial and complex steps in which abnormal gene expression plays an important role [3]. Intensive investigations over the last few decades have focused on the role of protein-coding genes in the pathogenesis of CRC. However, although only 1% of the human genome encodes proteins, 70% to 90% of the genome can be transcribed at some point during development to produce a large transcriptome of non-coding RNAs (ncRNA), 4-9% of which is transcribed to yield many short or long RNAs with limited protein-coding capacity [4]. Of these, the transcribed RNA molecules that are longer than 200 nucleotides and lack an open reading frame are defined as the lncRNAs [5]. LncRNAs are non-protein coding transcripts that are implicated in a number of important events, such as epigenetic, transcriptional, and post-transcriptional regulation [6]. LncRNAs exhibit unique profiles in various human cancers, reflecting disease progression and serving as a predictor of patient outcomes [7].

Many studies have reported that lncRNAs participate in various aspects of cell biology and potentially contribute to tumor development. Recently, the roles of lncRNAs as drivers of tumor suppressors and oncogenic function have been examined in diverse cancer types [8]. Over the past decades, multiple studies have shown that the CRC-related lncRNAs closely correlate with diverse biological processes involved in CRC progression through stimulating or inhibiting cell proliferation, apoptosis, differentiation, invasion and metastasis (Table 1). The lncRNAs are widely involved in the gene expression network at various levels, including chromatin modification, transcription, and posttranscriptional processing [9], For example, the lncRNAs Xist (X inactive-specific transcript) and HOTAIR (HOX Antisense Intergenic RNA) interact with chromatin remodeling complexes to induce local or global changes in chromatin packaging, leading to reduced target gene expression [10, 11]. LncRNAs can act as “miRNA sponges” and sequester miRNAs to inactivate these small regulatory RNAs and then to decrease the expression of miRNA target genes [12]. LncRNAs can also act as co-activators of transcription factors by interacting with RNA binding proteins and the interaction finally alters the localization and activity of the proteins [13]. Currently, the functions of lncRNAs remain poorly understood compared to those of short ncRNAs. Some lncRNAs have been detected in human body fluids by PCR, such as in plasma and urine [14, 15]. Here we will review the functions, mechanisms and biomarkers of CRC-associated lncRNAs from recently published papers, particularly focusing on the latest insights regarding progression of CRC. This information could be helpful to better understand the mechanisms and functions of CRC-related lncRNAs.

**Table 1:** The common abnormal expression of LncRNAs in CRC

LncRNA	Loci	Length	Biological Function
CCAT1	8q24.21	2628nt	Up-regulating c-Myc, promoting tumor cell proliferation and migration, function as a oncogene [61, 86]
CCAT2	8q24.21	340nt	Up-regulating c-Myc, promoting tumor cell proliferat ion a and migration, function as a oncogene [64, 87]
CCAT1-L	8q24.21	5200nt	Up-regulating c-Myc, promoting tumor cell proliferat ion and migration, function as a oncogene [63]
H19	11q15.5	6295nt	A dual function of oncogene and tumor suppressor gene [38, 41, 54, 88]
HOTAIR	12q13.13	2337nt	Recruiting PCR2 and LSD1 complexs to HOXD, silence HOXD, function as a oncogene [30, 89, 90]
MALAT1	11q13.1	8708nt	Regulating alternative splicing of the endogenous target gene, promoting cell proliferation, migration, and invasion function as a oncogene [15, 67, 69]
MEG3	14q32.2	1.6~1.8kb	Promoting the expression of P53 gene, inhibiting tumor growth, function as a tumor suppressor gene [42, 45, 91]
OCC-1	12q23.3	1139nt	Promoting cell proliferation, function as a oncogene [32]
PTENP1	9P21	3917nt	Binding to the regulatory region of miRNAs and the tumor suppressor gene- PTEN, function as a tumor suppressor gene [56]
UCA1	19P13.12	1441nt	Affecting cell growth and development, promoting tumor invasion, function as a oncogene [92, 93]
HULC	6p24.3	500nt	Regulating cell invasion and metastasis through competitively binding to miRNA. function as a oncogene [49]
Loc285194	3q13.31	2105nt	Regulating the expression of P53 and miRNA, inhibiting tumor growth, function as a tumor suppressor gene [29]
PCAT1	8q24	725nt	Promoting cell proliferation through association with PRC2, function as a oncogene [46, 47]
E2F4	16q21-22	~5000nt	Avoiding excessive cell proliferation [94]
CRNDE	16q12.2	*	Promoting cell proliferation, migration and invasion of colorectal tumor cells by acting as ceRNAs or interacting with chromatin-modifying complexes to regulate gene expression via epigenetic changes, function as a oncogene [83, 95-97]

* Related Content inaccurate; OCC-1: overexpression in colon carcinoma-1; UCA1: urothelial cancer-associated 1CRNDE: Colorectal neoplasia differentially expressed.

### What are the lncRNAs

**Figure 1:**
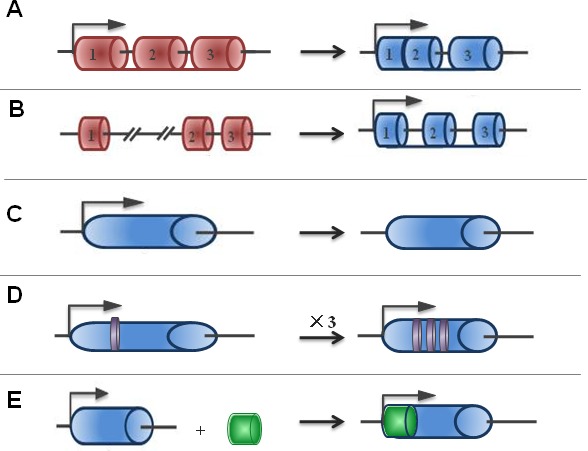
The origins of lncRNAs The possible origins of lncRNAs are divided into five broad categories: **A.** A protein-coding gene acquires structure interruption and is transformed into a lncRNA; **B.** As a result of chromatin reorganization, two untranscribed regions and another independent sequence region are juxtaposed and produce multiple exons of lncRNA; **C.** The duplication process of a non-coding gene by retrotransposition generates either a functional non-coding retrogene or a nonfunctional non-coding retropseudogene; **D.** Adjacent repeats of non-coding RNA originate from two tandem duplication events; and **E.** The insertion of a transposase gene gives rise to a functional lncRNA.

LncRNAs are RNA polymerase II (RNAPII) transcripts that can't encode for proteins [16, 17]. In the 1980s and 1990s, some lncRNAs were first identified, such as X-inactive specific transcript (XIST) and H19 were discovered by searching cDNA libraries for clones of interest [18, 19]. The size cut-off distinguished lncRNAs from small ncRNAs, such as microRNAs, small nucleolar RNAs (snoRNAs) and small interfering RNAs (siRNAs). There are a variety of different evolutionary scenarios for the emergence of functional lncRNAs, and they are roughly classified into five types (Figure 1): A) a protein-coding gene acquires structure interruption and is transformed into a lncRNA; B) through chromatin reorganization, two un-transcribed regions and another independent sequence region are juxtaposed and produce multiple exons of lncRNA; C) duplication of a non-coding gene by retrotransposition generates either a functional non-coding retrogene or a nonfunctional non-coding retropseudogene; D) the adjacent repeats of non-coding RNA originate in two tandem duplication events; and E) insertion of a transposase gene gives rise to a functional lncRNA [4]. Recent studies on lncRNAs in different species revealed that the size and function of various populations of RNA molecules differ. These lncRNAs include not only antisense, intronic, and intergenic transcripts but also pseudogenes and retrotransposons. According to the genetic view, these lncRNAs are in many forms: they can be very small or several hundred kilobases long; they may be spliced or unspliced; they can form linear or tertiary structures; they may have a poly-A tail; and they may interact with DNA, proteins, or other RNA molecules [20]. LncRNAs generally fall into five broad categories: (1) sense, when overlapping one or more exons of another transcript on the same strand; (2) antisense, when overlapping one or more exons of another transcript on the opposite strand; (3) bidirectional, when its expression and that of a neighboring coding transcript on the opposite strand are initiated in close genomic proximity; (4) intronic, when it is derived from an intron of a second transcript; and (5) intergenic, when it lies as an independent unit within the genomic interval between two genes [4]. This information on their positions will help scientists estimate the features of lncRNA. LncRNA may be located in the nuclear, chromatin, or cytoplasmic fractions of cells. The different localizations of lncRNAs suggest different biological functions. Increasing evidence indicates that lncRNAs may function as signals for transcription and decoys to transcription factors so that chromatin-modifying enzymes can be recruited to target genes and scaffolds to bring together multiple proteins to form ribonucleoprotein complexes [5, 21]. An additional biological function may include serving as a “sponge” to microRNAs [22]. Until now, the human genome and transcriptome sequencing databases demonstrated that half of the human genome is lncRNA. Despite their abundance, few lncRNAs have been studied and even fewer have been functionally characterized. This is in contrast to the small ncRNAs, such as siRNAs, miRNAs, and piRNAs, which are highly conserved and involved in transcriptional and post-transcriptional gene silencing through specific base pairing with their targets. While the numbers of characterized lncRNAs are increased, it is getting clear for their roles in stimulating or suppressing gene expression during cell differentiation and the development of human diseases [23, 24]. lncRNAs participate in epigenetic silencing, gene transcription and translation, the cell cycle and the apoptosis regulation process that is involved in DNA methylation, genomic imprinting, histone modification, microRNA interaction, protein interaction, and chromosomal instability [9, 25, 26]. Nevertheless, the biological functions of lncRNAs in the cancer cells are not well understood. To date, growing evidence suggests that cancer-associated lncRNAs, similar to protein-coding genes, may mediate oncogenic or tumor suppressing effects, and they may be a new class of cancer therapeutic markers [27, 28].

### LncRNAs in CRC development

To date, an increasing number of studies have shown abnormal expression of lncRNAs may inhibit tumor suppressor genes or cancer-promoting genes in the development of CRC. For example, loc285194 acts as a tumor suppressor but HOTAIR is the first identified lncRNA that plays a critical oncogenic role through epigenetic regulatory mechanisms [29, 30]. Gibb EA et al conjectured that abnormal expression of lncRNA was generally present in tumors [31]. Furthermore, recent studies have found different and unique expression of lncRNAs in CRC, and therefore lncRNAs could be new molecular markers in tumor diagnosis and treatment [32]. A few lncRNAs identified recently to be involved in the pathogenesis of CRC are showed in Table 1. Here, we will describe the status of several common CRC-associated lncRNAs according to their mechanisms in CRC.

### Epigenetic transcriptional regulatory lncRNAs

A general model for lncRNA-dependent gene-silencing mechanism is that lncRNAs first interact with chromatin to recruit histone-modifying enzymes, then induce chromatin modification and DNA methylation, and finally contribute to the epigenetic silencing of target genes. In this section, we will illustrate some epigenetic transcriptional regulatory CRC-associated lncRNAs.

### HOTAIR

HOX transcript antisense RNA (HOTAIR) can directly stimulate chromatin modifications related to tumor invasiveness and metastasis. HOTAIR plays a critical role in carcinogenesis through epigenetic regulatory mechanism, which was identified from a custom tilling array of the HOXC locus (12q13.13; ref. 2). HOTAIR is located in the 12q13.13 region, and its full length is 2.3 kb. It can form multiple double stem-loop structures to bind to the lysine-specific demethylase 1 (LSD1) and polycomb repressive complex 2 (PRC2) to form histone modification complexes [30], which stimulate the two complexes' integration with the corresponding specificity genomic loci and cause H3K27me3 (histone H3 tri-methylated at lysine 27) and H3K4me2 (histone H3 dimethyl Lys4) methylation, leading to HOXD silencing [33, 34]. In addition, Kailiang Zhang et.al found that HOTAIR also might accelerate cell cycle progression through EZH2 (predominant PRC2 complex component) but not LSD1 [35]. Clinical research showed that the expression levels of HOTAIR and the prognosis of patients are positively correlated in CRC, indicating that HOTAIR promotes metastasis, recurrence and prognosis of CRC [30, 36]. These findings suggest that HOTAIR plays an active role in modulating the cancer epigenome and may be an important target for the diagnosis and therapy of CRC.

### H19

H19, located on chromosome 11p15.5, close to the IGF II gene locus, is a maternally imprinted gene. It contains five exons and four introns with a length of 2.3 kb. The H19 gene does not encode protein but encodes a capped, spliced and polyadenylated 2.7 kb RNA. The imprinting of H19 is controlled by a region located 4 kb upstream of H19, which is defined as the H19 differentially methylated region (DMR) or imprinting control region (ICR). The H19 locus is subject to genomic imprinting and has often been used as a model for the study of epigenetic regulation [37, 38]. The oncogenic function of H19/miR-675 is to down-regulate tumor suppressor retinoblastoma (RB) in human CRC cells. Moreover, the proto-oncogene transcription factor c-Myc directly activates H19 in CRC implying that H19 is directly involved in the activation of the downstream gene expression of c-Myc [39]. However, studies have also indicated that H19 is a tumor suppressor *in vivo*. After H19 gene is knocked out, the capacity for tumor growth, invasion and metastasis is enhanced in murine models of colon tumorigenesis [40]. Therefore, abnormal expression of the H19 gene is related to the generation of CRC, and it dually functions as an oncogene and tumor suppressor gene through a variety of mechanisms [41].

### MEG3

Maternally expressed gene 3 (MEG3) is also an imprinted gene located at 14q32 that encodes an lncRNA. Multiple mechanisms contribute to the decreased expression of MEG3 in tumors, including gene deletion, promoter hypermethylation, and hypermethylation of the intergenic differentially methylated region. Furthermore, the treatment of human cancer cell lines with a methylation inhibitor results in MEG3 expression. The promoter region of MEG3 is rich in CpG dinucleotides. The methylation pattern of the 2.1 kb 5′-flanking region of MEG3 contains a total of 112 CpG dinucleotides upstream of the first exon, which is arbitrarily divided into four regions. Regions 1 and 4 contain functionally important sequences for gene expression [42]. The loss of MEG3 expression, its deletion from genomic DNA and the degree of promoter methylation are associated with aggressive tumor growth. These findings indicate that MEG3 may play a significant role as a novel long non-coding RNA tumor suppressor [43, 44]. Experimental data demonstrate that p53 is a target of MEG3 and that transfection of MEG3 induces a significant increase in p53 protein levels in human cancer cell lines [45].

### PCAT-1

The lncRNA prostate cancer-associated ncRNA transcript 1 (PCAT-1) is located in the chromosome 8q24 gene desert approximately 725 kb upstream of the c-MYC oncogene, and is involved in the progression of human prostate cancer. It has been shown that PCAT-1 is up-regulated in prostate tumor tissues and promotes prostate cancer cell proliferation *via* PRC2 [46]. Interestingly, unlike HOTAIR, PCAT-1 is repressed by PRC2. PCAT-1 functions chiefly as a transcriptional repressor by promoting the trans-regulation of genes preferentially involved in mitosis and cell division. PCAT-1 expression in CRC tissues is also significantly up-regulated compared with the matched normal tissues. There is a positive correlation between PCAT-1 expression and distant metastasis of CRC. The overall survival rate of the patients with high expression of PCAT-1 is significantly lower than those with low expression. In addition, multivariable Cox regression analysis identified PCAT-1 over-expression as an independent prognostic factor for CRC [47]. The molecular mechanisms underlying the altered expression of PCAT-1 in CRC remain unclear although it has been reported to be regulated by the PRC2 complex in prostate cancer cells.

### CCAT2

Studies showed that CCAT2 participates in epigenetic transcription by inducing chromosomal instability (CIN). CCAT2 is highly overexpressed in microsatellite-stable CRC and promotes tumor growth and metastasis. Studies have demonstrated CCAT2's ability to underlie CIN, manifested by the loss or gain of large portions or whole chromosomes, eventually resulting in aneuploidy, a characteristic trait of MSS CRCs. This is likely to occur through CCAT2's effects on downstream mediators, such as MYC and/or other WNT target genes. The direct correlation between MYC and MIR17HG in CIN has been previously demonstrated in that a transient excess of MYC activity could elicit genomic instability and carcinogenesis [48].

### LncRNA-RNA interactional lncRNAs

Recent work has highlighted a novel role for interactions between lncRNAs and RNA sequences. lncRNAs can function as competing endogenous RNAs (ceRNAs) to sponge miRNAs or as pseudogenes to serve as decoys for miRNAs, resulting in changes of target genes and the biological function of the miRNAs.

### HULC

Highly Up-regulated in Liver Cancer (HULC) is a typical example of “miRNA sponge”, which is located on the human chromosome 6p24.3 and consists of two exons. The length of its single intron is 500 bp. HULC is up-regulated in hepatocellular carcinoma (HCC) [49], but it is over-expressed not only in HCC but also in CRC that has metastasized to the liver. HULC is up-regulated in hepatic CRC metastasis, which is relevant to its null expression in normal tissues, primary CRC, or those cancers that metastasize to lymph nodes [50]. It contains a polyA tail and a particularly conserved miR-372 target site [49]. HULC binds to miR-372 and acts as a sponge to competitively inhibit miRNA from binding to a sense mRNA transcript. Since a recent study suggests that HULC may be involved in the process of CRC cell metastasis to the liver, it may become a new marker for evaluating the metastasis of CRC to the liver.

### Loc285194

Loc285194, also called LSAMP antisense RNA 3, is an lncRNA of more than 2 kb in length that consists of 4 exons located at osteo3q13.31. Loc285194 functions as a potential tumor suppressor. Study showed that loc285194 is a direct transcriptional target of p53 with two binding sites of miR-211in its exon 4. Loc285194 is also consistently down-regulated in colon tumor specimens compared with normal specimens and is a direct target of p53 through the negative regulation of miR-211 [29].

### H19

LncRNA is not only used as a model for the study of particular epigenetic regulation but also functions as miRNA sponges and as a miRNA precursor in CRC. For example, H19 can promote EMT progression and accelerate tumor growth by acting as competing endogenous RNA (ceRNA) for miR-138 and miR-200a in CRC [51]. Furthermore, H19 may form the precursor of miR-675 by an intracellular shearing action [52]. H19 expression is positively correlated with the level of miR-675, and miR-675 down-regulates RB expression [53]. The 3′-UTR of RB mRNA aligns with the sequence of mature miR-675, and the level of RB protein also appears to be negatively correlated with the levels of both H19 and miR-675 in human colon cancer cells [54]. These findings indicate that H19 can influence CRC progress through functioning as ceRNA or acts as precursor of miRNA, thus it can serve as a potential target for CRC treatment.

### PTENP1

The 3′UTR of PTENP1 is a pseudogene of PTEN. It can compete with PTEN for miRNA binding sites by binding to the regulatory region of miRNAs and can also be translated into the tumor suppressor protein PTEN. These findings suggest that the pseudogene participates in the regulation of homologous genes through non-coding RNA [55-57]. The proposed mechanisms remain controversial, and further investigation is required.

### LncRNA-protein interactional lncRNAs

It has been reported that many CRC-associated lncRNAs can directly bind to the target proteins or indirectly decrease or increase the expression levels of the target genes.

### CCAT

Colon cancer-associated transcript (CCAT) family members and some novel lncRNA transcripts map to the highly conserved 8q24.21 region that encompasses rs6983267. This region of interest has been shown to interact with MYC and the SNP rs6983267 at position 128.4 Mb, which influences MYC transcription [58-60]. It contains CCAT1 (Colorectal Cancer Associated Transcript 1), CCAT1-L (CCAT1, the Long isoform) and CCAT2 (Colorectal Cancer Associated Transcript 2). They participate in cell growth, cell cycle invasion and metastasis that is correlated to the nuclear transcription factor c-MYC.

### CCAT1

CCAT1 is 2628 nt in length with two exons corresponding to nucleotides 1-288 and 289-2612. The gene has an unusually long intron (approximately 9 kb). Its expression is tightly correlated with c-MYC [60]. Concretely, c-Myc directly binds to the E-box element in the promoter region of CCAT1 to regulate both the promoter activity and expression of CCAT1. CCAT1 is up-regulated in the majority of primary CRC tumors, precancerous polyps (adenomas), tumor-proximal colonic epithelia, lymph nodes, blood, and distant CRC metastases, but not in normal tissues [61]. Alaiyan B found that CCAT1 was up-regulated in both pre-malignant conditions and all disease stages in CRC [62]. Therefore, these novel biomarkers can be used for CRC screening, diagnosis, staging and novel therapies.

### CCAT1-L

CCAT-L is transcribed from 8q24.21, and its full length is 5200 nt. Specifically, it is transcribed in human CRC from a locus 515 kb upstream of MYC. CCAT-L located in the cell nucleus contains two exons and is polyadenylated. The over-expression of CCAT1-L can promote CRC tumorigenesis through the transcriptional regulation of MYC and the promotion of long-range chromatin looping. CCAT1-L localizes to its site of transcription and functions in the maintenance of long-range interactions between the MYC promoter. CCAT1-L interacts with CTCF and modulates it to bind to chromatin at the MYC locus [63]. These results revealed a novel relationship between the chromatin organization regulated by lncRNA and MYC expression in colon cancer.

### CCAT2

CCAT2, a 340-nt ncRNA, is transcribed from the MYC-335 region. CCAT2 expression is significantly higher in the CRC tissue than the adjacent mucosa. Moreover, microsatellite-stable (MSS) cancers have higher CCAT2 expression than microsatellite-unstable (MSI-H) tumors and the adjacent normal colon mucosa. CCAT2 enhances invasion and metastasis by influencing the MYC-activated miRNAs (miR-17-5p and miR-20) and the general activation of WNT signaling [64, 65]. Hui Ling et al. used RNA immunoprecipitation analysis to examine the physical interaction between CCAT2 and TCF7L2 by pulling down the RNAs that colocalized with TCF7L2 protein, and they found that endogenous CCAT2 could bind to TCF7L2 protein, increasing MYC expression in colon cancer cells [64].

### MEG3

The lncRNA MEG3 not only participates in methylation but also can influence the expression of the transcription factor p53. One study showed that p53 is a target gene of MEG3 andoverexpression of MEG3 leads to a significant increase in p53 protein levels in human cancer cell lines [45]. The study also found that MEG3 is absent from the human colon cancer cell lines HT29 and HT116, and an MEG3 isoform can cause a significant enhancement of p53-mediated reporter expression in HCT116 colon cancer cells [66].

### MALAT-1

Human cancer metastasis is associated with lung adenocarcinoma transcript 1 (MALAT-1), a long non-coding RNA that consists of more than 8,000 nt and is located on chromosome 11q13. It is highly expressed in many cancers and greatly related to metastasis. High expression of MALAT-1 is a strong predictor of poor prognosis in early cancers; however, some patients with low MALAT-1 levels also die during the 5-year follow-up period. MALAT-1 promotes cell migration, invasion, and metastasis [67]. Studies have found that a particular sequence (fragment 6918 nt-8441 nt), located at the 3′ end of MALAT-1 is correlated with several biological processes: cell proliferation, migration, and invasion of human colorectal malignant neoplasms. Therefore, this motif at the 3′ end of the MALAT-1 gene may be an important functional motif of MALAT-1. MALAT-1 is over-expressed in 50-80% of colon cancers, but exhibits low expression or no expression in normal tissues according to *in situ* hybridization [68]. MALAT-1 also accelerates CRC development and metastasis[69]. MALAT-1 is involved in mRNA splicing and nuclear paraspeckle function in cancer, and contributes to gene expression by regulating mRNA splicing, editing, and export [70]. Using CHIP combined with gene chip technology, Koshimizu TA and his colleagues found that there were transcription factor CREB binding sites in the MALAT-1 promoter region in neuroblastoma cell lines [71]. Therefore, MALAT-1 is a potential drug target and may facilitate the early diagnosis and establishment of a prognosis for neuroblastoma.

### The regulatory mechanisms of lncRNAs in CRC

**Figure 2:**
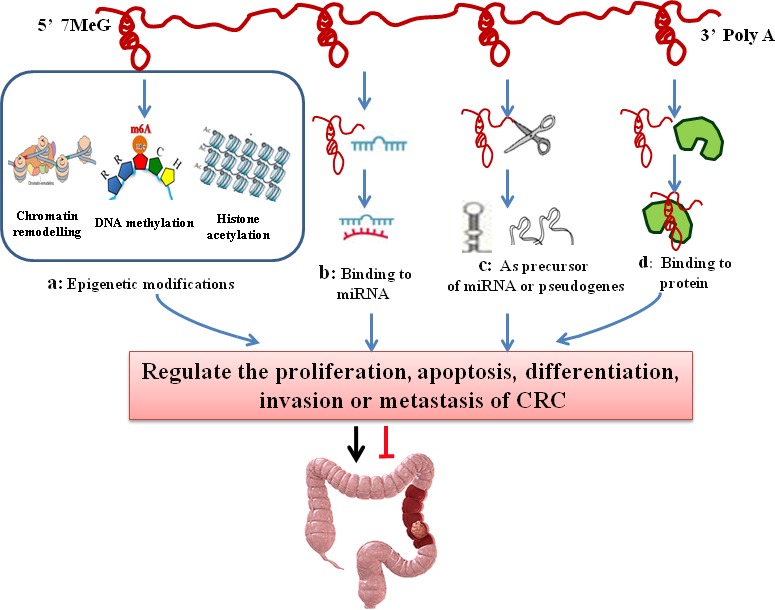
The regulatory mechanisms of lncRNAs in colorectal cancer LncRNAs regulate the proliferation, apoptosis, differentiation, invasion and metastasis of CRC in a variety of ways. **A.** LncRNAs induce chromatin modification, DNA methylation and histone acetylation and contribute to the epigenetic silencing or activation of target genes; **B.** LncRNAs regulate gene expression by binding to miRNAs and consequently preventing specific miRNAs from binding to their target mRNAs, thus regulating the expression of target mRNAs; **C.** LncRNAs act as pseudogenes or miRNA precursors; **D.** LncRNAs can serve as structural components or modulate protein activity or alter protein localization by binding to proteins.

LncRNAs are nearly involved in the whole life cycle of genes, from transcription to RNA splicing, degradation and translation. LncRNAs regulate gene expression in a variety of ways and have diverse mechanisms for regulating gene expression. lncRNAs have different regulatory mechanisms: they can act not only as the major transcription factor but also as one of the co-regulatory factors (co-regulators). Recent studies have demonstrated that lncRNAs are widely involved in the regulation of the gene expression network at epigenetic, transcriptional and post-transcriptional levels. The mechanism involved is closely associated with the genesis of CRC. Previous studies on lncRNAs have discovered that lncRNAs can activate or inhibit the expression of genes in a variety of dimensions, and the regulatory mechanisms are being uncovered. Now, we will focus on the mechanisms of its involving the development of CRC (Figure 2).

### Epigenetic modifications

LncRNAs play an important role in the epigenetic modifications of genes [72]. First, lncRNAs interact with a variety of chromatin modification enzymes, and then induce chromatin modification and DNA methylation. Eventually, they contribute to the epigenetic silencing or activation of target genes [73]. LncRNAs participate in allele silencing and the maintenance of epigenetic modifications during embryonic development. The targeting of epigenetic modifiers (EMs) by lncRNAs provides a much sought-after model to explain how EMs gains locus specificity and has since been suggested as a general mechanism for trans-acting lncRNAs [9, 74]. The major epigenetic modifications of CRC include DNA methylation, histone modifications, chromosomal modification (Figure 2a). The X-inactivation center (Xic) now serves as a typical model for understanding epigenetic transcriptional regulation by lncRNA. The typical example is X inactivation by the X-inactive-specific transcript (XIST/Xist) in mammals. Xist RNA directly binds to polycomb repressive complex 2 (PRC2), which is the epigenetic complex responsible for the trimethylation of histone H3 at Lys27 (H3K27me3), and targets PRC2 to Xi, silencing the whole chromosome [74]. Indeed, like Xist, the lncRNA HOTAIR also can bind to the PRC2, interact with chromatin remodeling complexes to induce heterochromatin formation in specific genomic loci, leading to reduced target gene expression [30, 36]. Those lncRNAs achieve their repressive function by coupling with histone modifying or chromatin remodeling protein complexes.

However, H19, which does not directly bind to PRC2 protein, is an imprinted and maternally expressed lncRNA that is spliced, polyadenylated, and exported into the cytoplasm where it accumulates to very high levels [19]. MEG3 is found to be abnormal CpG methylation in CRC, in which its expression is decreased. The hypermethylation in the MEG3 promoter region as well as the intergenic germ line-derived differentially methylated region (IG-DMR) would cause proliferation of cancer cells and blood vessels and then accelerate tumor metastasis and malignant [75]. CIN is another mode of epigenetic modification, which could be the underlying mechanism for CCAT-2 promoted CRC growth and metastasis. CCAT-2 may have effects on downstream mediators such as MYC and/or other WNT target genes [64]. Therefore, lncRNAs may be regulators of epigenetic mechanisms while actively participating in gene regulation. Despite of these exciting findings, the basic mechanisms how these lncRNAs work are still unknown.

### LncRNA-miRNA interaction

LncRNA can function as ceRNAs and “miRNA sponges” to antagonize their functions and lead to the de-repression of their endogenous targets, thereby imposing an additional level of post-transcriptional regulation. This lncRNA-miRNA interaction was first proposed in 2007 in a study that found the lncRNA IPS1 bound to the miRNA miR-399 and inhibited its ability to regulate PHO2 mRNA [76]. Recent evidence suggests that several CRC-related lncRNAs may also regulate gene expression by binding to miRNAs and consequently preventing specific miRNAs from binding to their target mRNAs (Figure 2b). This model more broadly suggests that lncRNAs, as well as other protein-coding mRNAs, may function as molecular “sponges” that binds miRNAs in order to indirectly control gene expression. A typical example of “miRNA sponges” is HULC, which is over-expressed in colorectal carcinomas that could metastasize to the liver [50]. HULC binds to miR-372 and acts as a sponge to competitively inhibit miRNA from binding to a sense mRNA transcript. Recently, researchers developed different databases to facilitate the study of lncRNA-miRNA interactions, such as miRcode (http://www.mircode.org), DIANA-LncBase (http://www.mircoma.gr/LncBase) and CHIPBase (http://deepbase.sysu.edu.cn/chipbase/) [77-79]. This will contribute to a better understanding of lncRNAs and miRNAs.

### LncRNA-protein interaction

LncRNAs can participate in global cellular behaviors by binding to specific proteins that contain nuclear transcription factors. Through lncRNA-protein interaction, lncRNAs can serve as structural components, modulating protein activity or altering protein localization (Figure 2d). As such, the mRNA stabilizing protein HuR accumulates in the cytoplasm when lincRNA-UFC1 is overexpressed, and the lincRNA-UFC1 binds directly to HuR, increasing the levels of β-catenin in HCC cells [80]. Guttman et al. studied 226 lncRNAs by RNA immunoprecipitation (RIP) analysis on the 28 types of chromatin complexes, and found that 12 kinds of complexes can bind to the studied lncRNAs. At least 30% (74/226) of lncRNAs can bind to one complex. However, lncRNA binding with multiple complexes is a widespread phenomenon [81]. In CRC, there are also many CRC-associated lncRNAs that directly bind to the target proteins or indirectly regulate the expression of the target genes. Indeed CCAT1-L, CCAT2, MEG3, Loc285194 and MALAT-1 promoter region have protein binding sites [29, 63, 64, 66, 71]. Taking CCAT2 as an example, Hui Ling et al used RIP analysis to examine the physical interaction between CCAT2 and TCF7L2 by pulling down RNAs colocalized with TCF7L2 protein, and they found that endogenous CCAT2 could bind to TCF7L2 protein, and then up-regulated MYC expression in colon cancer cells [64]. Thus, one lncRNA is possibly binds to different proteins butt the function may depend on its target protein.

### As pseudogenes

Pseudogenes are always regarded as “outcasts” of functional genes. However in recent years, they have drawn increasing attentions. For example, the stability of mRNA is increased by pseudogenes which is transcribed by homologous genes through generating a shortened non-coding segments (Figure 2c). It is also found that pseudogene can influence the expreession of homologous genes with transcribing antisense sequence. In CRC-associated lncRNA, the 3′UTR of PTENP1 is a tumor suppressor pseudogenes, which can bind to the regulatory region of miRNAs but also bind to the tumor suppressor gene PTEN. Therefore, the 3′UTR of PTENP1 causes the decline of PTEN mRNA expression, and also can be translated into the tumor suppressor protein (PTEN). These findings suggest that the pseudogene participates in the regulation of homologous genes through non-coding RNA [55-57]. However, the underlying mechanisms remain controversial and the further investigation is required.

### As precursor of miRNA

In addition to binding miRNA, several studies have demonstrated that lncRNAs can regulate gene expression by acting as miRNA precursors (Figure 2c). Fox example, H19 as the precursor of miR-675, can decrease the level of RB [52]. The 3′-UTR of RB mRNA aligned with the sequence of mature miR-675 and the level of RB protein also appears to be negatively correlated with the levels of both H19 and miR-675 in the human colon cancer cells [53]. This intriguing hypothesis indicates that H19 is derived from miR-675 to regulate CRC development.

### Clinical biomarkers of lncRNAs in CRC

lncRNAs and their deregulation have been described across both solid and hematological malignancies. Similar to miRNAs, lncRNAs have both cell and tissue specificity and regulatory function. In most instances, evidence has relied on differences in transcript expression levels between disease- and non-disease-associated states. Ye Hu et.al used a lncRNA-mining approach, established a six-lncRNA signature. They demonstrated three dysregulated lncRNAs, AK123657, BX648207 and BX649059 were necessary for efficient invasion and proliferation suppression in CRC cell lines. This study provides an efficient classification tool for clinical prognosis evaluation of CRC [82]. Reliable molecular markers, analyzed in non-invasively obtained surrogate samples, may assist in the selection of the best possible diagnosis and treatment for individual cancer patients. These markers have various types, including diagnostic markers, prognostic markers, and therapeutic markers [28].

In recent years, there are few studies have investigated whether lncRNAs can serve as effective diagnostic and prognostic biomarkers for CRC. HOTAIR and CRNDE, for example, are upregulated in neoplastic tumor tissue and in the blood of CRC patients [83, 84]. Moreover, HOTAIR is associated with patients' overall survival, and its high levels are correlated with TNM stage and histologic grade. These data suggest that HOTAIR levels in the blood may serve as potential surrogate prognostic marker in CRC [84]. With the development of experimental techniques, Kam Y. et al applied CCAT1-specific peptide nucleic acid (PNA) based molecular beacons (TO-PNA-MB) to serve as a diagnostic probe for *in situ* (human colon biopsies) detection of CRC. *In situ* hybridization of selected TO-PNA-MB in human CRC specimens, CCAT1 expression was detected in all (4/4) subjects with pre-cancerous adenomas, and in all (8/8) patients with invasive adenocarcinoma tumors. These results suggest that CCAT1 TO-PNA-MB is a powerful diagnostic tool for the specific identification of CRC [85]. Another lncRNAs, CCAT2 may also be served as a diagnostic biomarker since its expression is significantly higher in the tumor tissues as compared with the adjacent mucosae of 215 CRC and 94 paired non-neoplastic mucosal specimens obtained from patients from four different geographical regions [64]; Therefore, lncRNAs transcripts in the cancer tissues and in the plasma of patients are promising biomarkers for CRC.

## CONCLUSIONS

Along with small RNAs and proteins, lncRNAs have broad applications in CRC diagnosis and treatment. The unique expression or over-expression of lncRNAs in CRC may be used to develop new tumor markers. However, since lncRNA research is still in its infancy, there are several unresolved issues using lncRNA as clinical biomarkers for the diagnosis and treatment of CRC. Furthermore, only a limited number of CRC-related lncRNAs has been well characterized. Therefore, a systematic identification of lncRNAs and a better understanding of their mechanisms are needed to facilitate their applications to the diagnosis and treatment of CRC. Recently, with a growing evidence for the role of tumor-specific lncRNA and the site-specific action of cis-acting lncRNAs in tumor development, researchers have begun to develop lncRNA-targeting drugs.

Although the molecular mechanisms of lncRNAs are not well understood,the lncRNAs summarized in Table 1 may provide a glimpse of the full range of lncRNA functions in cancer. Further studies are needed to focus on the mechanisms and regulation of lncRNAs in normal and cancer cells. With the development of research and technology, lncRNAs will facilitate the diagnosis and treatment of CRC in the near future.

## Abbreviations

CRC: colorectal cancer
lncRNAs: long non-coding RNAs
XIST: X-inactive specific transcript
MALAT-1: metastasis associated lung adenocarcinoma transcript 1
HOTAIR: human homeobox transcript antisense RNA
PRC2: polycomb repressive complex 2
CCAT1: colorectal cancer associated transcript 1
CCAT2: colorectal cancer associated transcript 2
HULC: highly up-regulated in liver cancer
CIN: chromosomal instability
c-Myc: cellular homologue of avian myelocytomatosis virus oncogene
CREB: cAMP response element-binding protein
PCAT-1: Prostate Cancer Associated Transcripts 1
HER2: Human Epithelial Growth Factor Receptor 2
snoRNAs: small nucleolar RNAs
siRNAs: small interfering RNAs.
